# Complementary Detection of Zero-Value Insulators Based on the Fusion of Infrared Images and Electric Field Intensity Features

**DOI:** 10.3390/jimaging12100505

**Published:** 2026-10-12

**Authors:** Wei Liu, Jiang Yang, Jun Su, Tao Yang, Teng Wang, Tao Wang

**Affiliations:** 1 Guangyuan Power Supply Company of State Grid Sichuan Electric Power Company, Guangyuan 628000, China; 2 College of Electrical and Electronic Information, Xihua University, Chengdu 610039, China

**Keywords:** zero-value insulator, infrared image, YOLOv8, electric field intensity, feature fusion

## Abstract

To address the electrical safety hazards caused by missing zero-value insulators in high-voltage transmission lines, this study proposes a complementary detection method based on the fusion of infrared images and electric field intensity features. Infrared images of insulator strings are acquired using a UAV-mounted infrared thermal camera, and the surface temperature of each insulator is extracted via YOLOv8 to construct a temperature anomaly factor. Meanwhile, a three-dimensional electric field model of the insulator string is established using COMSOL Multiphysics 6.2 to extract the axial electric field intensity and calculate the electric field deviation. These two types of features are combined into a two-dimensional vector, and the Minimum Covariance Determinant (MCD) is used to estimate robust mean and covariance, from which a robust Mahalanobis distance is calculated to generate a fusion score for zero-value insulator identification. A three-level classification criterion is established, categorizing insulators as normal, suspicious, or zero-value. Experimental results demonstrate that this method effectively exploits the complementarity of infrared and electric field features, achieving the accurate identification of zero-value insulators, with a precision of 93.8%, zero false-negative and false-positive rates, and a suspicious rate of 6.2%. The method is validated to be highly accurate and robust.

## 1. Introduction

Transmission lines are the core infrastructure of power systems, and their safe and stable operation is directly related to national energy supply and socio-economic development [1,2,3]. As the key electrical insulation and mechanical support components of transmission lines, the performance state of insulators directly determines the insulation level of the whole line [4,5,6]. With the increase in voltage level and operation life, insulators will inevitably be subjected to the coupling effect of multiple factors such as lightning shock, surface pollution, and mechanical stress in a long-term complex field environment, resulting in deterioration and failure [7,8,9]. Among them, the zero-value insulator is one of the most common and most destructive defects in transmission line operation. When the zero-value insulator loses its insulation ability, the voltage distribution of the insulator string will be seriously distorted, causing other normal insulators to bear excessive voltage, which can easily lead to flashover fault [10]. In addition, zero-value insulators easily leads to vicious accidents such as insulator shattering, string detachment, and conductor drop. In severe cases, they will lead to large-scale power outages, seriously threatening the safe and reliable operation of power systems [11].

Therefore, it is of great significance to identify and replace zero-value insulators in time and accurately for ensuring the safe and stable operation of power system. At present, the diagnosis of zero-value insulators mainly relies on two kinds of methods based on thermodynamic characteristics and electromagnetic characteristics. Representative methods are surface temperature detection based on infrared thermal imaging and anomaly identification based on electric field distribution characteristics.

Infrared thermal imaging technology is based on the physical principle that insulator defects lead to a decrease in insulation resistance and an increase in leakage current, generating Joule heat. It measures the temperature distribution of the insulator surface in a non-contact way to identify the defect state, which effectively avoids the interference to the operation of high-voltage lines. Ref. [12] uses active infrared thermography with thermal excitation to detect the impact damage by analyzing temperature differences, followed by image processing and a comparison with the ultrasonic C-scan results. However, it relies on external thermal excitation and mainly single-source temperature information, with limited validation under complex field conditions and automated defect diagnosis. Ref. [13] analyzes the infrared measurement accuracy under dynamic conditions by modeling the effects of platform motion and optical-system temperature variations. It uses calibration and temperature compensation to reduce measurement errors. However, it mainly focuses on space-based infrared measurement, and does not consider complex factors such as outdoor environmental interference, target emissivity variations, or multi-source information fusion. In addition, it is difficult to fully reflect the electrical insulation state inside the equipment only by relying on the surface thermal field distribution, which limits the upper limit accuracy of its detection [14,15,16].

On the other hand, the electric field distribution characteristics of insulators can directly reflect their electrical insulation properties. Through the Finite Element Method (FEM) combined with professional simulation software such as COMSOL, three-dimensional models of insulators [17,18] can be established and their spatial electric field distribution can be accurately calculated. When the insulator fails at zero value, the electric field distribution around the insulator will be obviously distorted. This method can visually present the spatial variation of electric field intensity and potential abnormal areas, which is helpful to deeply reveal the working state and fault evolution mechanism of equipment. Compared with infrared detection, electric field features have significant advantages such as strong physical directivity and less influence by the meteorological environment when used for fault diagnosis of high-voltage equipment [19]. However, the pure electric field simulation method relies heavily on the complex prior modeling work, and requires extremely precise equipment structural parameters and material properties. In practical engineering applications, it is difficult to match the complex and changeable physical boundary conditions in real time.

In summary, in the existing zero-value insulator detection methods, infrared thermal imaging and electric field analysis are usually independent. Although these methods have certain applicability in specific scenarios, they are prone to the missed detection of concealed faults or the misjudgment of environmental interference under complex working conditions. The above limitations highlight the need to develop thermal-electric multimodal feature fusion detection methods. By integrating infrared temperature information with electric field characteristics, it can not only improve the accuracy of equipment fault detection, but also provide multi-dimensional information support for insulator state diagnosis in complex scenarios.

In order to solve the above problems, a complementary zero-value insulator detection method based on YOLO infrared temperature extraction and COMSOL electric field simulation is proposed in this paper, which aims to fundamentally improve the detection accuracy and anti-interference ability of zero-value insulators. This paper constructs the concept of electric field-temperature complementary detection, which treats electric field distortion as the primary criterion and temperature anomaly as the auxiliary validation, and makes full use of the non-correlation and complementarity of two kinds of physical quantities to establish a multi-dimensional fusion criterion matrix. Specifically, the YOLO algorithm is used to accurately extract the insulator and its temperature characteristics in infrared images, and the joint analysis of electric-field features generated for the modeled scenarios and thermal features obtained from infrared images is carried out, which significantly improves the detection efficiency and recognition accuracy of zero-value insulators.

The core innovations of this study are summarized as follows: The methodological contributions of this study are as follows. First, a piece-level electric-field deviation is defined relative to a physically constrained trend along the insulator string and paired with a piece-level thermal anomaly feature. Second, the two features are represented jointly in a two-dimensional space; the Minimum Covariance Determinant method estimates a reference center and covariance, and a robust Mahalanobis distance quantifies the joint deviation. Third, the joint score is combined with separate feature checks to assign one of three outcomes: normal, suspicious, or zero-value. The suspicious outcome explicitly indicates that further inspection is required.

The paper is structured as follows: Section 2 focuses on infrared image analysis and temperature extraction, Section 3 addresses the analysis of electric field intensity, and Section 4 deals with data fusion and the construction of a zero-value detection criterion.

## 2. Infrared Image Analysis and Temperature Extraction

### 2.1. Image Acquisition and Processing

In this study, an unmanned aerial vehicle platform equipped with infrared thermal imaging camera is used to conduct the non-contact inspection of insulator strings of transmission lines to obtain high-resolution infrared images. The inspection height and shooting angle of the UAV are adjusted reasonably according to different voltage levels and circuit structure specifications, ensuring that each insulator string is fully captured within the field of view with sufficient clarity and resolution to accurately identify local temperature rise anomalies. Thermal images were acquired during UAV inspections. The specific thermal-camera model cannot be recovered from the available acquisition records; therefore, camera-specific performance specifications are not asserted in this study. The retained images have a resolution of 640 × 480 pixels and are stored as JPG files. The image dataset contains 3000 annotated insulator units. Of these, 2400 images were used for training, 300 for validation, and 300 for testing.

The acquired infrared images need to be pre-processed before entering the analysis process, including removing ambient noise, enhancing image contrast, and cropping irrelevant background areas to improve the salience of the insulator target. These preprocessing steps not only help to eliminate the interference factors in the image acquisition process, but also provide high-quality input for subsequent target detection based on YOLOv8 [20,21,22,23,24], significantly improving the detection accuracy and algorithm stability. Through this method of combining UAV inspection and image preprocessing, the rapid, efficient, and accurate monitoring of transmission line insulator strings can be realized, providing a reliable data basis for zero-value insulator identification.

### 2.2. Temperature Extraction of Insulator Infrared Picture Based on YOLOv8

**Figure 1:**
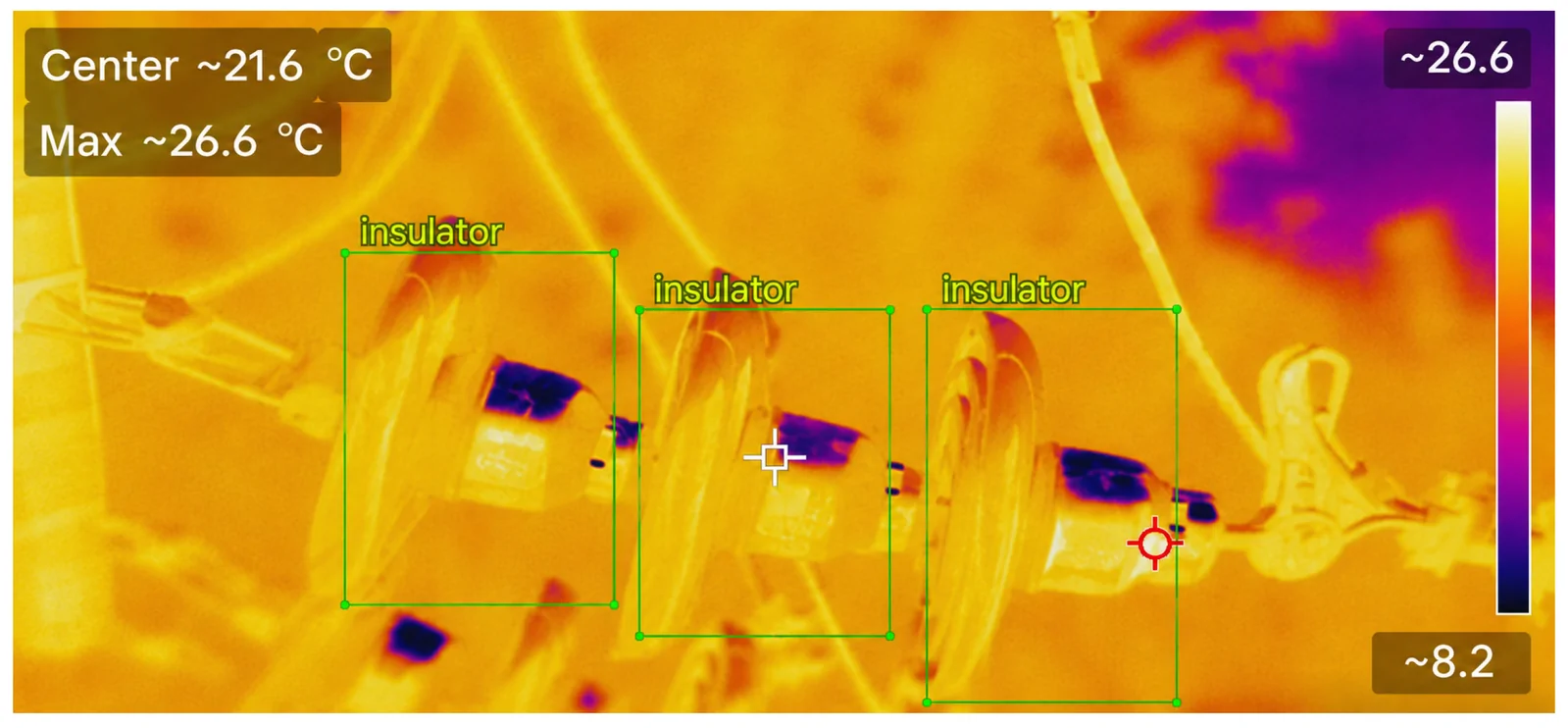
Annotation example.

An unmodified YOLOv8 detector was used to locate individual insulator units in the thermal images. Predicted coordinates were subsequently mapped to the original image coordinates for piece-level analysis. After the image preprocessing is completed, based on the YOLOv8 target detection algorithm, the insulator target is accurately located, and the suspected degradation area is extracted based on the YOLOv8 target detection algorithm. First, for the infrared image data set of transmission lines, the bounding box and suspected deterioration parts of each insulator are manually marked, and divided into the training set, verification set, and test set according to the ratio of 8:1:1. The labeling example is shown in Figure 1. Subsequently, the training samples are augmented by data enhancement methods such as rotation, flip, brightness adjustment, and Gaussian noise addition to enhance the robustness of the model for different shooting angles and environmental conditions. In the training stage of YOLOv8 model, transfer learning is performed using pre-training weights to accelerate convergence and improve detection accuracy, while appropriate learning rate, batch size, and iteration number are set to optimize the performance of the model on infrared insulator images.

After training, the model can be rapidly and accurately applied to newly acquired images to identify individual insulators, frame temperature anomaly areas, and output corresponding position information, providing a reliable data basis for subsequent electro-thermal characteristic analysis and multi-physics field fusion.

YOLOv8 is employed to automatically detect and locate each individual insulator unit in the infrared image and to obtain the corresponding region of interest (ROI). It should be noted that the bounding box generated by YOLOv8 is used only for the localization of an individual insulator unit rather than being directly regarded as the effective region for temperature calculation. Since the rectangular bounding box may contain background regions, particularly when the insulator string is inclined, background pixels within the ROI are excluded through subsequent image processing. Therefore, the temperature features are calculated only from the valid pixels belonging to the insulator region after background exclusion, thereby reducing the influence of the surrounding environment, such as the sky, vegetation, towers, and conductors, on temperature extraction. Let the infrared $I\in R^{H\times W\times 3}$ image be, and the set of bounding boxes of each insulator output by the model is as follows:

(1) $$B={{\left\{\left.\left({x_{1}}^{i},{y_{1}}^{i},{x_{2}}^{i},{y_{2}}^{i},{P_{cls}}^{i}\right)\right\}\right.}_{i=1}}^{N}$$

where $\left({x_{1}}^{i},y_{1}^{i}\right)$ and $\left({x_{2}}^{i},{y_{2}}^{i}\right)$ are the coordinates of the upper left and lower right corners of the *i*-th bounding box, respectively; ${P_{cls}}^{i}$ is the classification probability; and N is the number of insulators detected in the image. For the bounding box area of the *i*-th insulator $R_{i}$,

(2) $$R_{i}=T\left[{y^{i}}_{1}:{y^{i}}_{2}:{x^{i}}_{1}:{x^{i}}_{2}\right]$$

Take the statistics of the temperature in this area:

(3) $${T^{i}}_{mean}=\frac{1}{\left|R_{i}\right|}\sum_{\left(x,y\right)\in R_{i}}T\left(x,y\right)$$

(4) $${T^{i}}_{\max}=\underset{\left(x,y\right)\in R_{i}}{\max}T\left(x,y\right)$$

(5) $${T^{i}}_{\min}=\underset{\left(x,y\right)\in R_{i}}{\min}T\left(x,y\right)$$

where ${T^{i}}_{mean}$ represents the average temperature of the region, ${T^{i}}_{\max}$ is the highest temperature of the region, ${T^{i}}_{\min}$ is the lowest temperature of the region, and $\left|R_{i}\right|$ is the number of pixels in the region. Through these statistical values, the average temperature, the highest temperature, and the lowest temperature of each insulator can be obtained, which provides a data basis for subsequent analysis.

(6) $$T_{i}=\frac{1}{N_{i}}\sum_{p\in i}t_{p}$$

where $T_{i}$ is the temperature of the *i*-th insulator piece; $t_{p}$ is the pixel temperature value; and $N_{i}$ is the number of pixels within the detection box. After enhancing the abnormal temperature sensitivity by weighted averaging, a temperature sequence can be obtained:

(7) $$T=\left[T_{1},T_{2},T_{3},\ldots ,T_{n}\right]$$

The YOLOv8 model is used to train the infrared images of insulators. In order to evaluate the detection accuracy of different target detection models for insulator defects, the accuracy (P), recall (R), and average accuracy (*mAP*) are selected in this paper to evaluate the detection performance of the model. The calculation formula of the above indicators is as follows:

(8) $$\mathrm{Precision}=\frac{TP}{TP+FP}\times 100\%$$

(9) $$\mathrm{Recall}=\frac{TP}{TP+FN}\times 100\%$$

(10) $$mAP=\frac{\sum_{i=0}^{N}AP_{i}}{N}$$

where *TP* represents true positive, that is, the number of targets correctly detected by the model; *FP* stands for false positives, that is, the number of non-targets that the model incorrectly identifies as targets; *FN* stands for false negatives and represents the number of actual positive examples not detected by the model; *N* represents the number of categories of the dataset; and AP*i* represents the recognition accuracy of the *i*-th category.

**Figure 2:**
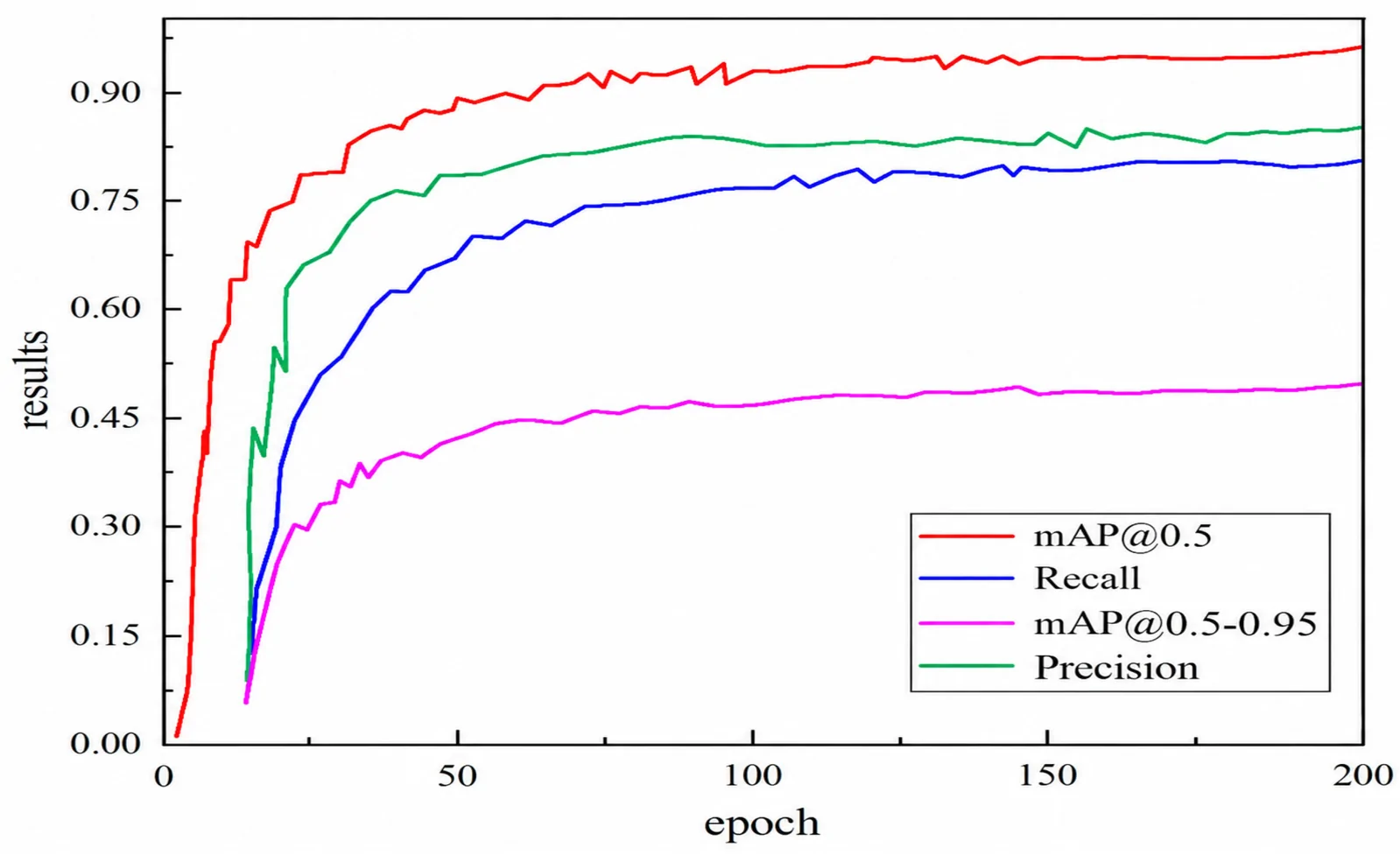
Training results.

During model training, the initial learning rate is set to 0.01, the final learning rate is set to 0.01, the number of working threads is 8, the optimizer selects SGD, the weight attenuation coefficient is set to 0.005, the Batch Size is uniformly used 16, the momentum is set to 0.937, and the training cycle is 200 rounds. The training results are shown in Figure 2.

**Figure 3:**
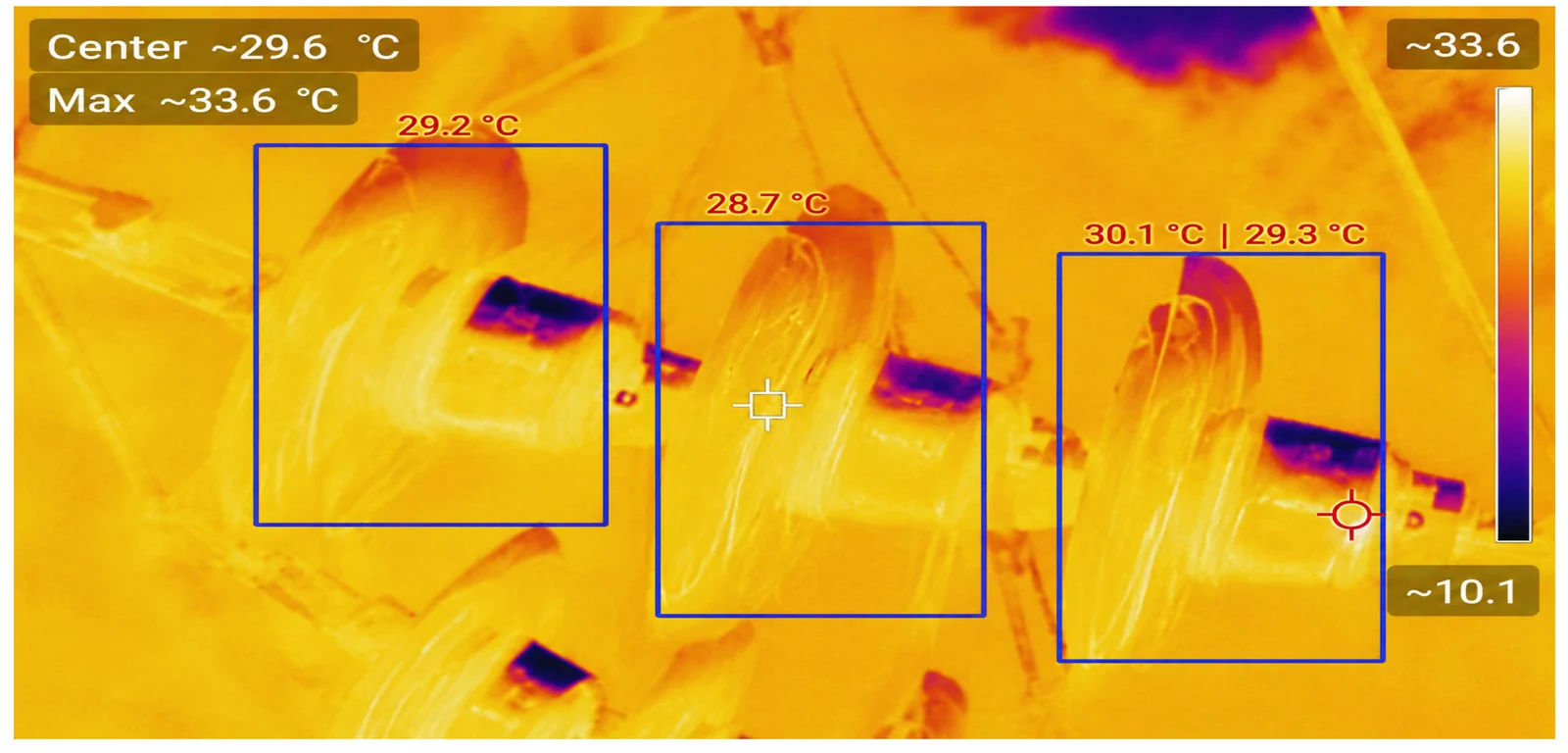
Output results.

The YOLOv8 model is used to detect the target of infrared images, and the bounding box of each insulator is accurately located. Rendered thermal images were used for object localization. Piece-level temperatures were calculated from the corresponding source temperature data after mapping the detection coordinates to the source-image coordinates. Display-color adjustment was not used to convert the HSV brightness into temperature. Statistical analysis is performed on the area within the bounding box of each insulator output by YOLOv8, and the average temperature, maximum temperature, and minimum temperature are calculated. It enables the batch processing of infrared images within a folder, automatically generating Excel spreadsheets and annotated charts containing temperature data for each insulator. To improve efficiency, the method supports the batch processing of all infrared images in the folder and generating visual annotations: draw each insulator bounding box in the image; mark the average temperature above the box; and each picture corresponds to an Excel file, which saves all insulator temperature information. The method realizes the automatic batch processing of a large number of insulator infrared images without manual intervention one by one, significantly improves the data processing efficiency, and can be applied to offline analysis in laboratory and on-line detection scenarios in substations and transmission lines at the same time. The processed image can visually present the temperature distribution characteristics along the surface of the insulator string, providing a reliable data base for the subsequent low/zero-value insulator identification and positioning based on abnormal temperature rise. Typical processing results are shown in Figure 3.

The automatic identification and positioning of insulators is realized by YOLOv8, which effectively avoids the subjective error caused by manual ROI delimitation. The algorithm can handle complex background and multi-angle images, thus improving the accuracy of temperature extraction. In addition, it supports the feature of batch processing, which greatly improves the inspection efficiency.

## 3. Electric Field Strength Analysis

### 3.1. Insulator String Model Establishment

**Figure 4:**
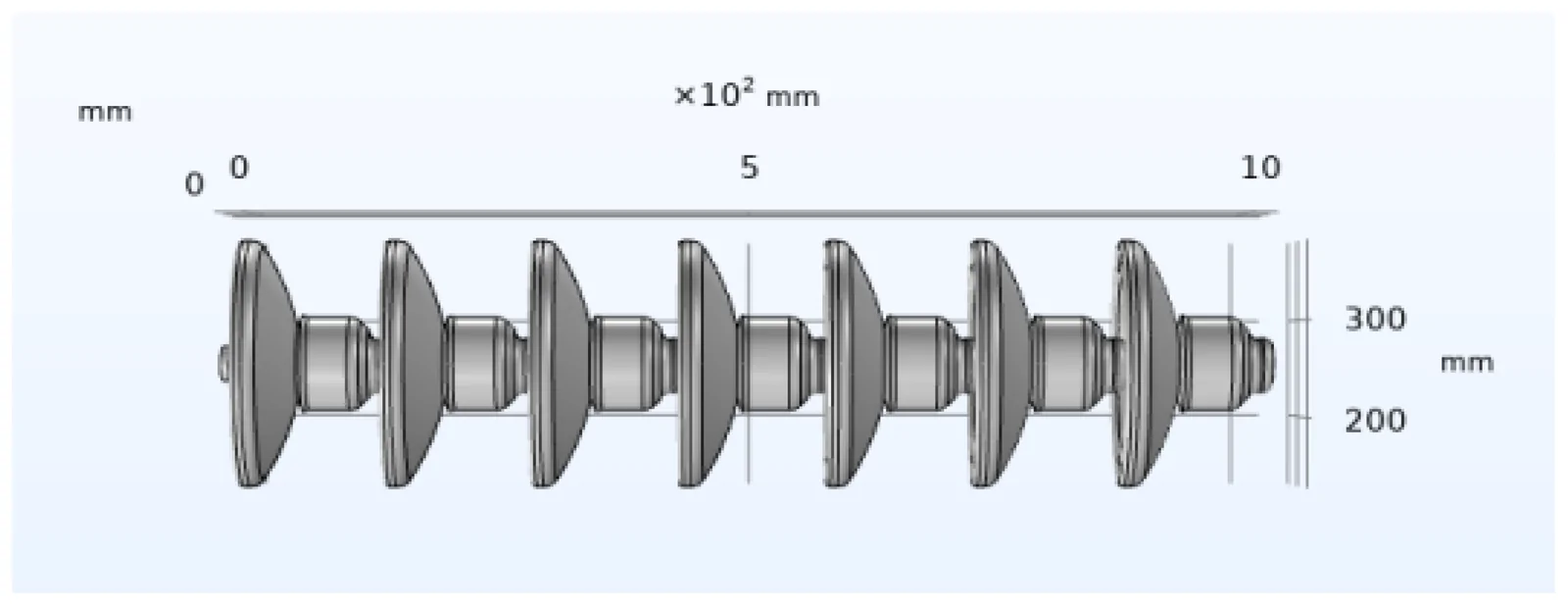
Simulation model diagram.

In this study, the U70BP-160 porcelain insulator string is taken as the research object, and its three-dimensional simulation model is established by using COMSOL Multiphysics 6.2 software, as shown in Figure 4. The insulator string consists of seven pieces of insulators, including the porcelain plate, steel cap, steel foot, and adhesive. Among them, the height of the porcelain plate is 155 mm, the diameter is 255 mm, the diameter of the steel foot is 18 mm, and the diameter of the steel cap is 96 mm. In the model, the insulators are numbered 1 to 7 from bottom to top. The COMSOL simulation in this study is conducted under normal atmospheric conditions. The purpose of the simulation is to establish the reference electric-field distribution characteristics of the insulator string under a standard operating environment and to investigate the variation in the electric-field distribution caused by changes in the electrical state of individual insulator units. Therefore, additional environmental disturbances are not introduced into the present simulation. The obtained electric-field distribution should be regarded as a reference characteristic under normal atmospheric conditions rather than as a complete representation of all possible field environments.

In the setting of electrical boundary conditions, the bottom No. 1 insulator is connected to a high-voltage wire, and its steel foot voltage is set to a phase voltage of 63.5 kV (corresponding to a voltage level of 110 kV); the steel cap of the top 7 insulator is grounded at a potential of 0 V. To simulate the environment of the insulator in actual operation, the model is placed in a dry air domain with a size of 10 m × 20 m × 10 m, assuming that the air is at normal temperature and pressure without flow. For power frequency AC lines, the voltage distribution of insulator strings is mainly characterized by capacitance, and its specific distribution law is determined by the relative permittivity of the constituent materials. In this study, the relative dielectric constant of air is taken as 1, the relative dielectric constant of porcelain plate is taken as 6, and the relative dielectric constant of steel foot and steel cap is set as 10^5^, so as to accurately reflect the dielectric characteristics of each component in electric field.

**Figure 5:**
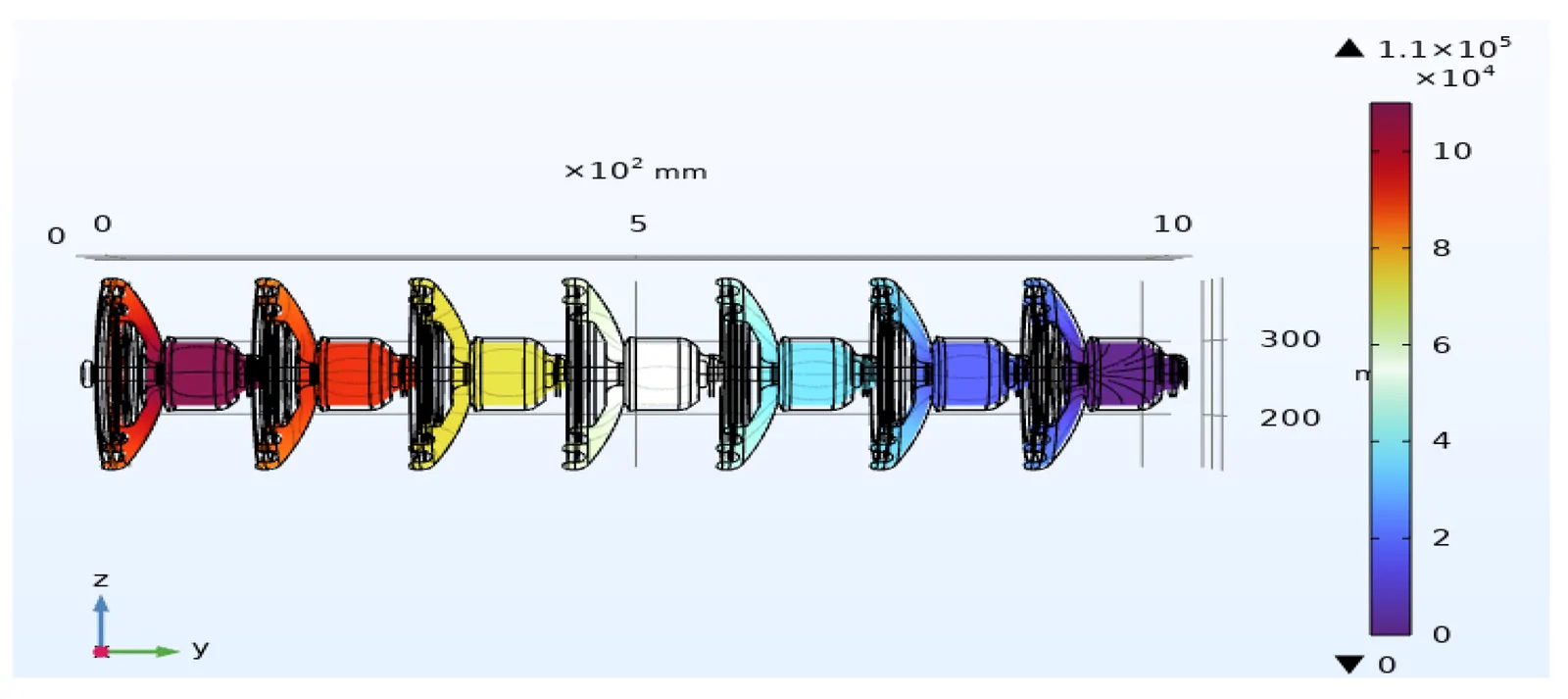
Insulator string potential diagram.

Through numerical calculation and post-processing analysis, the global potential distribution cloud diagram of the insulator string under the normal state is obtained, as shown in Figure 5, which reflects the distribution law of voltage in space. In the normal state, the potential exhibits a continuous gradient distribution along the axial direction of the insulator string, and smoothly transitions from the high voltage terminal to the ground terminal. The potential difference distribution of each insulator is mainly determined by its geometric parameters and dielectric characteristics, which conforms to the series distribution characteristics of capacitors.

### 3.2. Extraction and Processing of Electric Field Strength Data

On the selection of eigenvalues, there are many candidate schemes such as the average value, maximum value, and weighted integral. Considering that the concentration of the electric field distortion induced by the zero-value insulator in the local area is much greater than the overall offset—that is, the electric field strength of the zero-value sheet itself decreases significantly, while the electric field strength of the specific local area of the adjacent normal sheet climbs sharply—the maximum value can be used as the characteristic quantity to capture this local abnormal signal to the greatest extent. The mean value may mask the key local distortion features due to the smoothing effect, which is not conducive to subsequent anomaly detection. Accordingly, the raw feature value of the piece-level electric field intensity $E_{i}^{raw}$ for the *i*-th insulator piece is defined as the maximum value of the electric field magnitude along its corresponding sampling path and the neighboring region.

(11) $$E_{i}^{raw}=\underset{p\in \Omega_{i}}{\max}\left|E\left(p\right)\right|$$

where $\Omega_{i}$ is the effective sampling domain of the *i*-th insulator piece, specifically including all discrete points on the sampling path and their adjacent nodes extending outward along the normal direction of the surface. The setting of this range takes into account both the grid resolution and the variation scale of electric field gradient along the surface, and effectively avoids the numerical fluctuation caused by the poor single-point grid quality or local singularity.

The original electric field sequence extracted by the above steps is of the order of 10^2^ kV/m, which differs from the infrared temperature sequence (of the order of 10^1^ °C) by several orders of magnitude on the numerical scale. In order to eliminate the dimensional difference, the Min–Max linear normalization transformation is performed independently on the electric field sequence of each string of insulators according to Equation (12).

(12) $$E_{i}=\frac{E_{i}^{raw}-\min(E^{raw})}{\max(E^{raw})-\min(E^{raw})}$$

where $\min(E^{raw})$ and $\max(E^{raw})$ are the minimum and maximum values of the raw eigenvalues of the electric field for the seven insulator pieces in the current insulator string, respectively. After transformation, $E_{i}\in [0,1]$.

## 4. Data Fusion and Zero Detection Criterion Construction

### 4.1. Data Standardization and Feature Extraction

The infrared temperature and electric field intensity of insulator operating states have different physical dimensions and numerical ranges. If the two kinds of original data are directly fused, the significant difference in numerical scale will lead to certain features occupying an improper dominant position in the discrimination. Therefore, the two kinds of raw data need to be processed at the feature level before data fusion, and converted into comparable anomaly metrics. In this paper, two kinds of characteristics are constructed to realize the unified quantification of data characteristics: the electric field deviation degree measures the distortion degree of electric field strength of a certain insulator relative to the theoretical normal distribution, and the temperature anomaly factor measures the deviation degree of heat generation level of a certain insulator relative to the normal state. The above two together constitute the input features of the subsequent fusion criteria.

#### 4.1.1. Electric Field Deviation

Keep the distance between the monitoring path and the insulator axis at 300 mml the electric field intensities of the insulator are numbered sequentially from the high-voltage end to the grounding end as $E_{1},E_{2},\ldots ,E_{i}$. A number of simulations and measurements show that the resultant electric field of a normal insulator string is distributed in an asymmetric U-shape along the string, being the highest at the high voltage end, gradually decaying to the middle part, and rising slightly at the ground end. When a certain insulator in the string is a zero-value insulator, the voltage at both ends of this insulator will approach zero, which will cause the surrounding electric field to significantly deviate from the normal distribution mode and form an obvious depression on the U-shaped curve.

In order to quantify this distortion, it is necessary to first determine the normal electric field trend line of the $E_{i}^{trend}$ string, that is, assuming that all the insulators of the string follow the electric field value distribution under normal conditions, and then calculate the electric field deviation degree of the *i*-th insulator based on the trend line:

(13) $$\Delta E_{i}=\frac{\left|E_{i}-E_{i}^{trend}\right|}{E_{i}^{trend}+\varepsilon }$$

where $\varepsilon =10^{-6}$ is used for numerical stability, and does not affect the physical meaning of the deviation degree. This index is dimensionless and can reflect the distortion degree of the electric field distribution. Because the insulation resistance of a zero-value insulator drops significantly, its surrounding electric field will be significantly different from the normal trend, and, correspondingly, $\Delta E_{i}$ will increase significantly.

However, under different voltage levels, insulator string types, and detection distances, there are differences in the numerical range and attenuation rate of the spatial electric field of normal insulator strings, and the position and number of zero-value insulators in actual measurement are unknown. If the trend is constructed according to a fixed ratio based on a certain electric field value, when the electric field value is exactly zero, the whole trend line will shift, resulting in a wide range of misjudgment.

In order to solve the above problems, a constrained quadratic fitting method based on the consensus principle of Random Sample Consensus (RANSAC) is designed to adaptively determine the normal electric field trend line. Because of the U-shaped distribution of the electric field of the insulator string, it can be approximated as a quadratic function with the opening facing upwards: $E(i)=ai^{2}+bi+c(a>0)$. Unlike the conventional RANSAC procedure that relies on random sampling when dealing with large datasets, the number of insulators in a single string is limited in the present application. Therefore, the consensus principle is retained, while the candidate subsets are directly enumerated instead of being generated by stochastic sampling. For a string containing N insulators, m = $\left[0.6\;N\right]$ points are used to form each candidate normal set, consistent with the original subset-size setting. The total number of candidate subsets is $C_{N}^{m}$, which remains limited for the insulator-string sizes considered in this study and allows all candidate combinations to be evaluated with a low computational cost. The proposed consensus fitting strategy is also applicable to short insulator strings. For example, for a seven-unit insulator string, seven electric-field measurement points are still sufficient for constructing and evaluating candidate quadratic models, since at least three points are required to determine a quadratic model. Because candidate subsets are deterministically enumerated rather than randomly sampled, the fitting procedure can systematically examine the eligible subsets even when the total number of insulator units is small. However, as the number of units decreases, the redundancy available for robust model estimation is correspondingly reduced. Therefore, the reliability of the consensus model depends on retaining a sufficient number of normal units, and its robustness may decrease when abnormal units account for a large proportion of a short insulator string.

**Figure 6:**
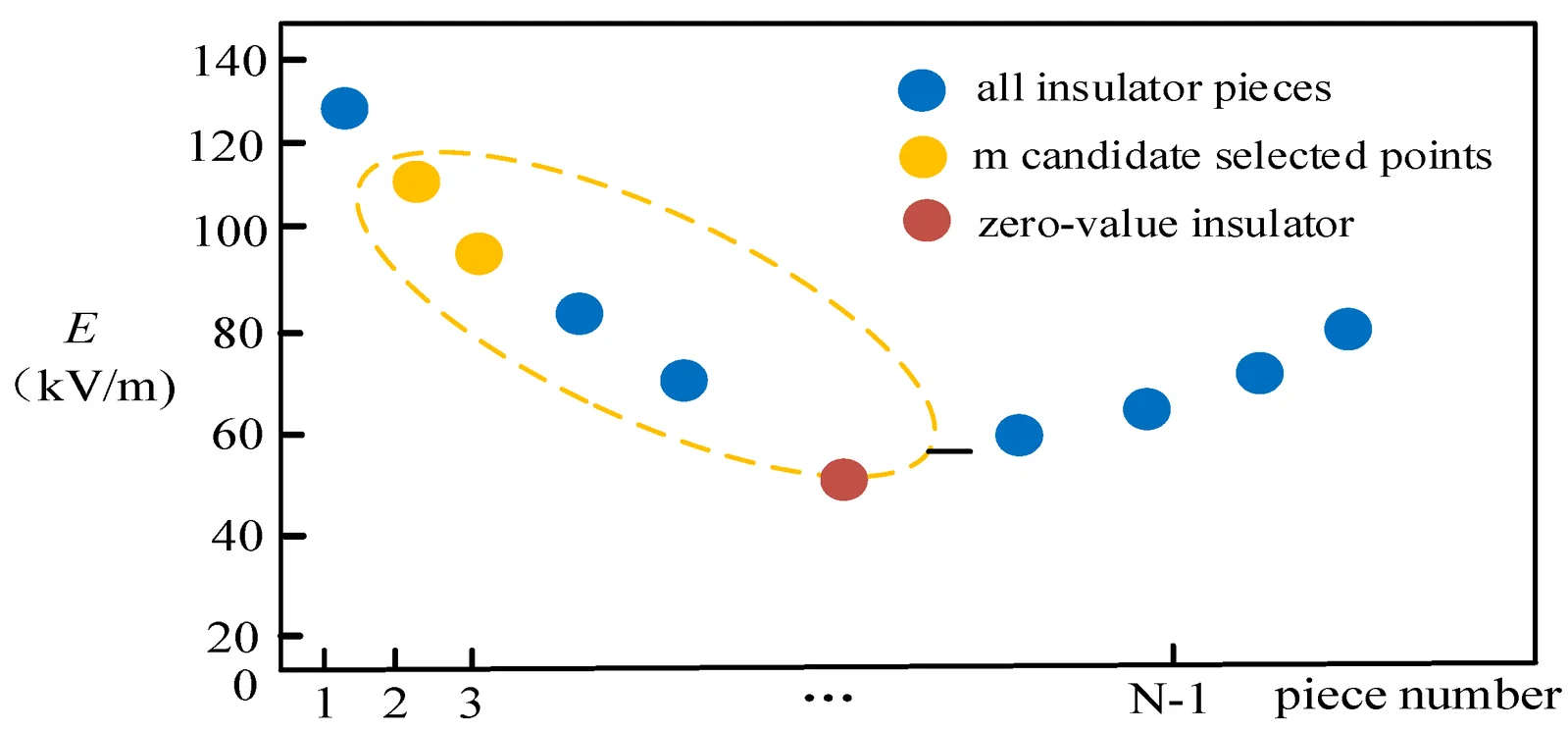
Random sampling curve plot.

**Figure 7:**
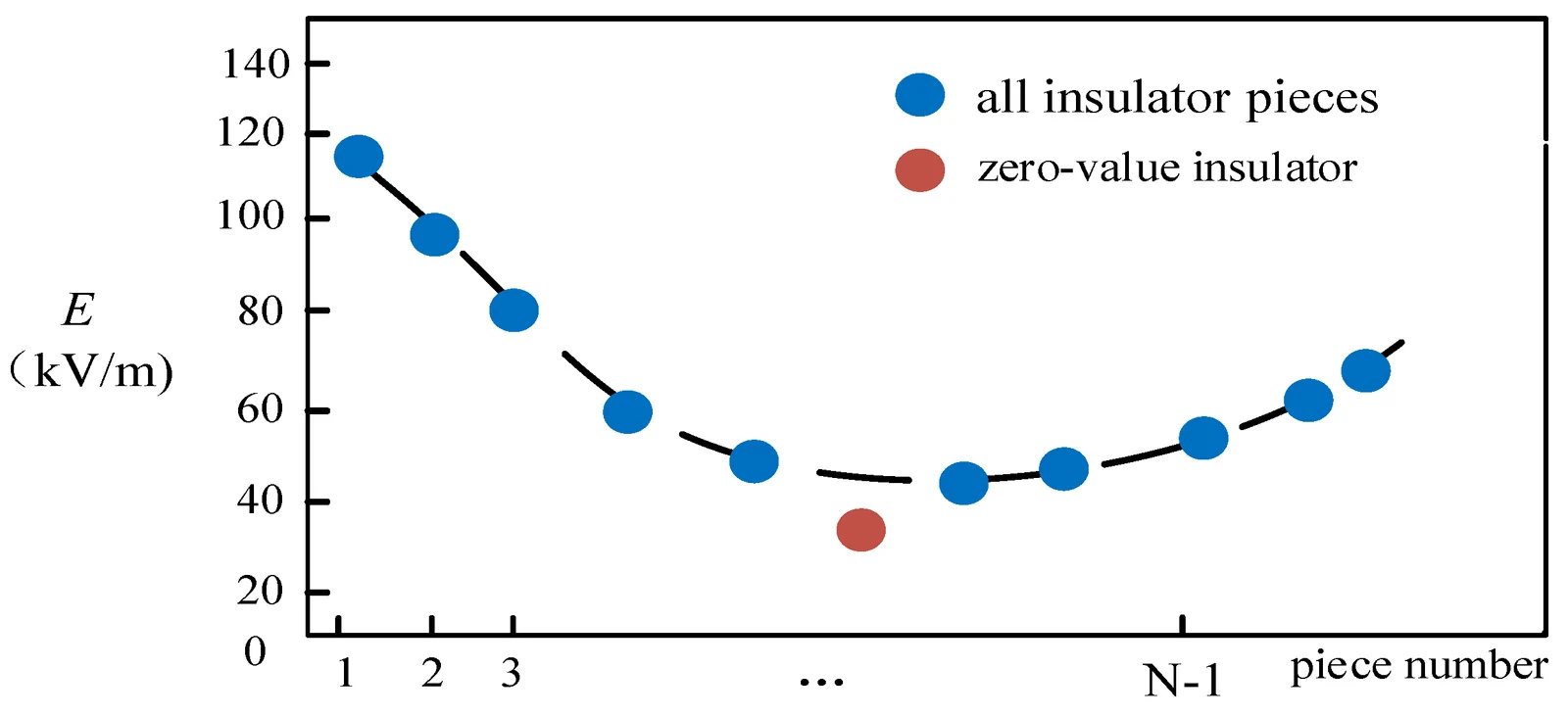
Schematic diagram of quadratic curve fitting.

For each candidate normal set, a quadratic curve is fitted and checked to determine whether it satisfies the following three physical constraints: (1) the opening faces upward; (2) the minimum position falls within the interval of the second to the N-1-th insulators; and (3) the fitting value at the high-voltage end is higher than that at the grounding end. This procedure combines the physical distribution characteristics of the insulator-string electric field with the consensus screening mechanism, thereby avoiding the dependence of the fitted trend on a single preset reference point. The candidate subset selection and quadratic curve fitting processes are shown in Figure 6 and Figure 7.

For each candidate curve satisfying the above physical constraints, the deviation of every insulator in the full string relative to the fitted curve is calculated, and the number of inliers whose deviation falls below the threshold is counted. After traversing all candidate point combinations, the model with the highest inlier count is selected as the optimal hypothesis; if multiple hypotheses have the same inlier count, the one with the smallest median deviation is selected. In this way, the candidate curve that is most consistent with the overall normal electric field distribution can be identified without introducing randomness into the fitting process.

The rationale for this method is that the electric field values of normal insulator pieces consistently follow the inherent U-shaped distribution trend, whereas zero-value insulators exhibit obvious local deviations from this trend because of electric field distortion. Therefore, the abnormal points generally show a lower consistency with the normal electric field pattern and are excluded from the optimal consensus set. Accordingly, a quadratic curve is finally refitted using the electric field data of all normal pieces within the identified optimal normal-piece set to obtain the final standard trend line $E_{i}^{trend}$.

#### 4.1.2. Temperature Anomaly Factors

After the infrared image is detected by the YOLOv8 target detector and the temperature is extracted, the surface temperature of each insulator is obtained, and the ambient temperature $T_{i}$ is recorded at the same time. The dielectric loss $T_{amb}$ and leakage current of the insulator at the operating voltage can make it warmer than the environment. Due to the extremely low voltage sharing, the heat generation of a zero-value insulator is weak, and the temperature rise $\Delta T_{i}=T_{i}-T_{amb}$ is significantly smaller than that of a normal insulator. For this reason, the structural temperature anomaly factor is defined as follows:

(14) $$\alpha_{i}=\max(0,\min(1,1-\frac{\Delta T_{i}}{\tau }))$$

where τ=2 °C, is the temperature rise threshold parameter, as the temperature rise in normal insulators is generally higher than 2 °C, while the temperature rise in zero-value insulators is generally lower than this threshold. When the temperature rise is greater $\alpha_{i}=0$ than or equal to 2 °C, it indicates that the temperature of the insulator is not abnormal; on the contrary, the smaller the temperature rise $\alpha_{i}$, the closer it is to 1, which indicates a higher degree of temperature abnormality. The temperature abnormality factor can compress the temperature information to the [0,1] interval, which visually shows the abnormal degree of insulator temperature.

The temperature-difference threshold adopted in this study was selected with reference to the infrared characteristics of zero-value insulators reported in Ref. [4]. In that study, when a zero-value insulator was located at the first, fourth, and seventh positions of an insulator string, the differences between the maximum and minimum temperatures were approximately 1.4 °C, 1.7 °C, and 2.8 °C, respectively, and the maximum temperature difference was identified as a characteristic quantity for zero-value insulator diagnosis. Based on these reported temperature-difference characteristics and the experimental conditions considered in this study, 2 °C was adopted as the reference threshold for characterizing a significant infrared thermal anomaly. This value is used as a thermal-feature criterion in the proposed electric–thermal complementary diagnostic framework rather than as a universal threshold for independently determining zero-value insulators. Its applicability should therefore be further verified or recalibrated when the operating conditions, insulator type, or environmental conditions differ substantially from those considered in this study.

### 4.2. Fusion Model Construction

With two dimensionless indices such as $\Delta E_{i}$, the electric field deviation degree, and $\alpha_{i}$, the temperature anomaly factor, it is necessary to integrate the two kinds of heterogeneous characteristics into a unified discriminant index for the detection of zero-value insulators. In this paper, the two kinds of features are regarded as coordinate points in two-dimensional feature space, and the zero-value problem is transformed into a two-dimensional outlier detection problem.

The electric field deviation degree of the *i*-th insulator and the temperature anomaly factor are constituted as a two-dimensional eigenvector $x_{i}={\left[\Delta E_{i},\alpha_{i}\right]}^{T}$, and are expressed in two-dimensional space. For normal insulators, their eigenvalues are usually close to zero, constituting a point cloud close to the origin; however, the eigenvalues of zero-value insulators increase significantly, so they will be far away from the normal point set in two-dimensional space. Formulating the problem as the detection of outliers in two-dimensional space is advantageous to consider the joint effect of electric field and temperature characteristics simultaneously.

#### 4.2.1. Robust Mahalanobis Distance

In order to quantitatively characterize the degree of deviation between the temperature distribution along the surface of insulator string and the normal state, appropriate statistical metrics should be introduced in order to transform the subjectively perceived temperature anomaly into an objective and comparable numerical criterion and distinguish the normal point from the abnormal point. In this paper, the Robust Mahalanobis Distance (RMD) method is adopted. The Mahalanobis distance is defined as follows:

(15) $$D_{M,i}=\sqrt{{\left(x_{i}-\hat{\mu }\right)}^{⊤}{\hat{\sum }}^{-1}(x_{i}-\hat{\mu })}$$

where $x_{i}$ is the feature vector of the ii-th insulator, and $\hat{\mu }\in R$ means the value of the normal eigenvector and its $\hat{\sum }\in R^{2\times 2}$ covariance matrix. The physical meaning of the Mahalanobis distance is that the distance of the direction with a large normal fluctuation is reduced, and the direction with a small fluctuation but an abnormality is amplified by the distance, so that the weighted fusion is automatically realized without artificially specifying the fusion weight.

The Mahalanobis distance can adaptively scale the deviation of each direction according to the covariance structure of the normal point set, so that the direction with a large fluctuation allows a larger deviation, while the direction with a small fluctuation is more sensitive to anomalies, thus realizing the adaptive fusion of the electric field deviation degree and temperature anomaly factor without artificially setting the weight. And, in the two-dimensional feature space, the feature vectors of normal insulators $\hat{\mu }$ are usually distributed near the normal point cloud $\hat{\sum }$ described by the robust mean and the robust covariance, and the deviation degree is within the allowable fluctuation range along all directions, so the corresponding Mahalanobis distance is small; however, the eigenvector of zero-value insulator will deviate from the center position of normal point cloud due to at least one obvious anomaly of feature, especially in the direction of the small covariance, which leads to the significant increase in its Mahalanobis distance.

#### 4.2.2. Robust Parameter Estimation

In practical calculation, the presence of zero-value insulators will affect the overall distribution of normal point sets. If μ and ∑ are calculated directly from the full string data, they may be contaminated by zero-value samples. That is, the abnormal samples may shift the mean toward the anomalous region and expand the covariance ellipse, thereby reducing the Mahalanobis distance of abnormal samples and resulting in the masking effect. Therefore, the Minimum Covariance Determinant (MCD) algorithm is used for robust estimation. For the condition in which only a small number of abnormal samples are present, the robust Mahalanobis distance obtained from MCD estimation can effectively suppress the influence of abnormal points and improve the reliability of zero-value insulator detection.

The algorithm automatically searches for a subset containing at least $\left[0.75\times N\right]$ insulators, such that the determinant of the covariance matrix of this subset is minimized; that is, the corresponding point cloud is the most compact. The robust mean and robust covariance matrix are then calculated based on the identified normal subset.

In practical operation, zero-value insulators are relatively rare abnormal events, and the simultaneous occurrence of multiple zero-value units within the same insulator string is even less common. Therefore, the statistical model in this study mainly considers the condition in which only one or a small number of zero-value insulators are present in a string. For the seven-unit insulator string considered in this study, the MCD subset contains five samples. When one zero-value insulator occurs, six normal insulators remain, and, even when two zero-value insulators occur simultaneously, five normal insulators are still available to form an uncontaminated subset. Under these conditions, the robust location and covariance can be estimated without necessarily incorporating the abnormal samples.

When the number of simultaneous zero-value insulators further increases, the proportion of abnormal samples may exceed the applicable range of the current robust estimation. For example, when three zero-value insulators occur in a seven-unit string, only four normal insulators remain, which is fewer than the five samples contained in the MCD subset. In this case, at least one abnormal sample may be included in the candidate subset, and the estimated robust mean and covariance matrix may consequently be affected, weakening the suppression of the masking effect. Therefore, high-proportion multiple-defect conditions are not included in the main statistical assumption of the present model, and dedicated robust estimation strategies for such conditions require further investigation.

#### 4.2.3. Fusion Score

Assuming that a normal sample approximately obeys a binary Gaussian distribution, the square of its Mahalanobis distance $D_{M}^{2}$ obeys a chi-square distribution with 2$\chi^{2}(2)$ degrees of freedom. Take the 97.5% quantile as the abnormality determination threshold, and define the normalized fusion score as follows:

(16) $$S_{i}=\frac{D_{M,i}}{\tau }$$

where $S_{i}\leq 1$ indicates that the insulator unit is within the normal fluctuation range, and $S_{i}>1$ indicates a statistically significant anomaly.

### 4.3. Zero-Value Insulator Zero Detection Criteria

Based on electric field deviation, temperature anomaly factor, and fusion score, this paper proposes a three-level classification discrimination strategy, which divides the insulator status into normal, suspicious, and zero-value, so as to meet different detection sensitivity and decision-making requirements in transmission inspection.

#### 4.3.1. Adaptive Feature Anomaly Flags

The Mahalanobis distance can judge whether a certain insulator deviates statistically significantly from the normal point cloud, but the statistical significance is not equivalent to physical anomaly. Because when the feature distribution of the normal subset is too concentrated, minimal feature differences between normal slices may also be amplified to statistically significant deviations, resulting in false positives. To avoid such misjudgments, a feature anomaly flag based on the absolute dispersion of the median is introduced, and a physical bottom line is set separately for each feature.

**Figure 8:**
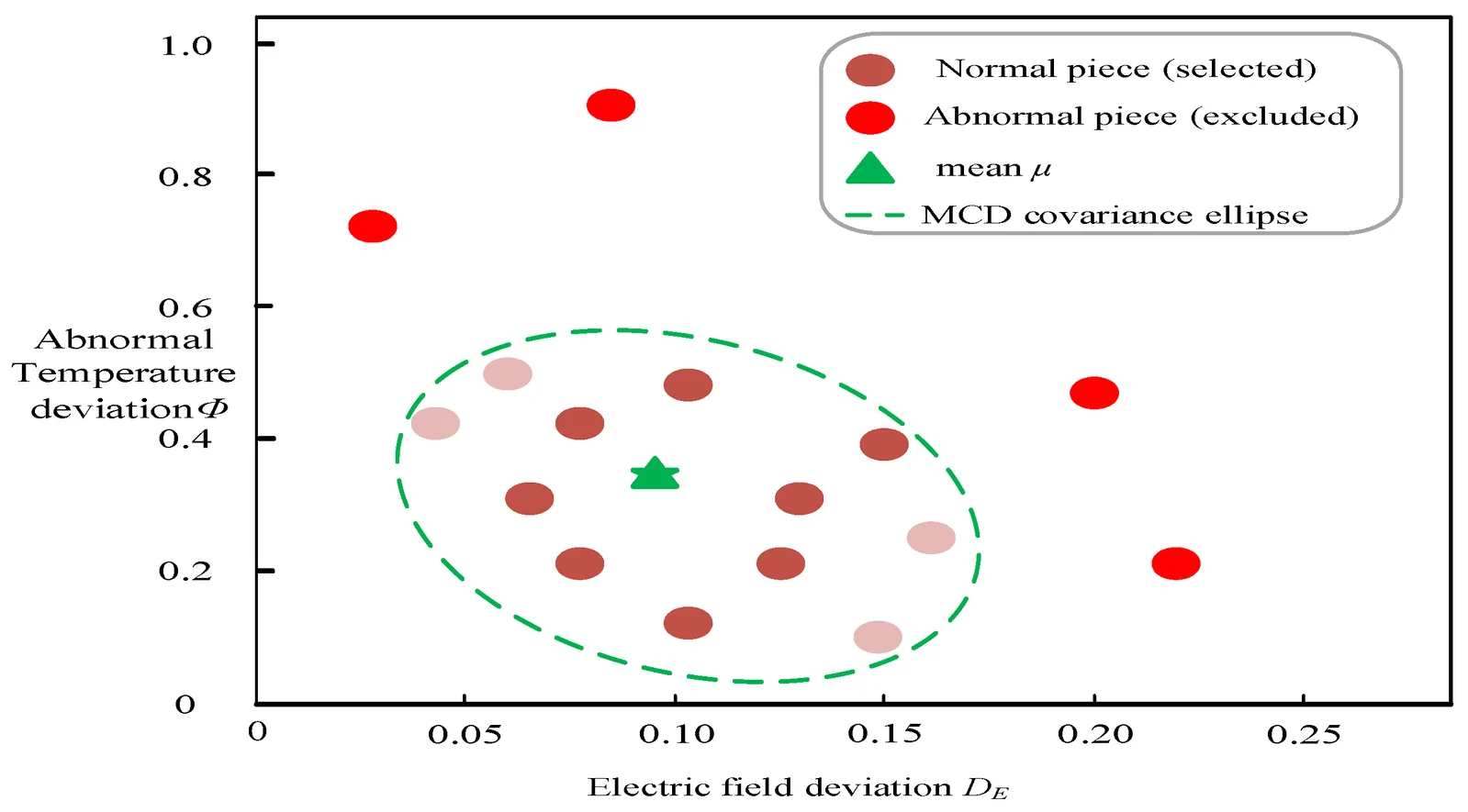
MCD robust estimation.

**Figure 9:**
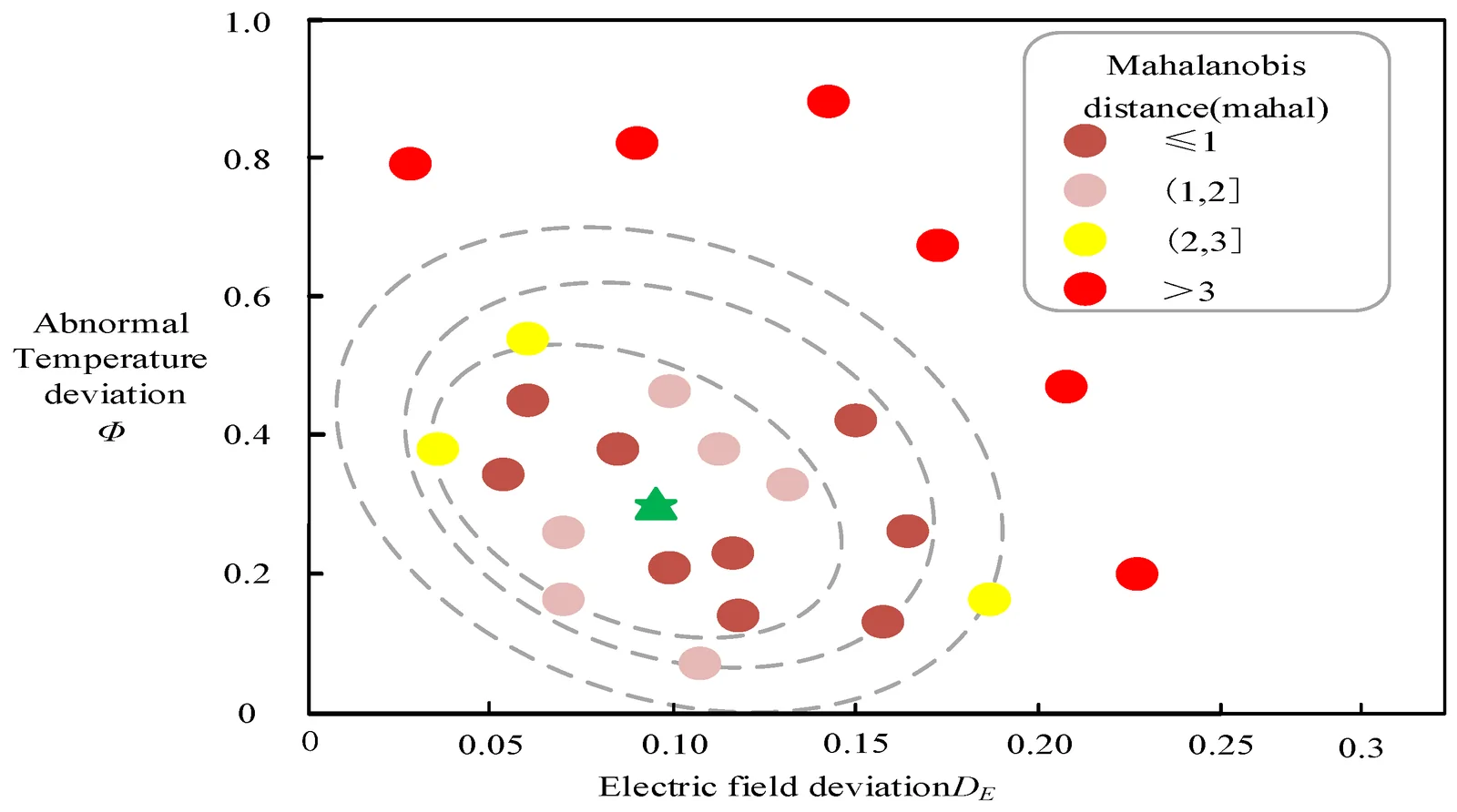
Mahalanobis distance and fusion score.

For the normal subset selected by the MCD method, the median electric-field deviation and the median temperature anomaly factor are calculated. Their dispersion is estimated using the Median Absolute Deviation (MAD). Compared with the standard deviation, MAD is substantially less sensitive to individual outliers and can characterize the feature distribution of normal insulator units more robustly. An insulator unit is considered to have an abnormal electric-field feature when its electric-field deviation exceeds both the statistical threshold and the physical lower bound. Similarly, its temperature feature is considered abnormal when the temperature anomaly factor exceeds both the statistical threshold and the physical lower bound and remains above the absolute baseline of 0.15. The corresponding results are shown in Figure 8 and Figure 9.

This two-condition design ensures that the statistical threshold remains consistent with the normal fluctuation range of the current insulator string, while the physical lower bound prevents minor fluctuations from being incorrectly classified as abnormalities when the normal subset is excessively concentrated. For example, when the overall condition of an insulator string is good and the electric-field deviations of all units are concentrated around 0.02, the dispersion estimated by MAD may be as low as 0.005. In this case, an electric-field deviation of 0.03 may be statistically higher than the corresponding threshold for a normal insulator unit. However, because it does not reach the physical lower bound of 0.10, its electric-field anomaly flag remains 0 and the unit is not incorrectly classified as abnormal.

#### 4.3.2. Grading Criteria for Fusion Scoring and Feature Markers

Based on the fusion score and characteristic abnormal flag, this paper formulates a three-level classification criterion, and classifies the insulator state into three categories: normal, suspicious, and zero.

The determination of zero-value insulators needs to meet three conditions at the same time: the fusion score exceeds the statistical anomaly threshold of 1—that is, the Mahalanobis distance exceeds the chi-square quantile of 97.5%, and the electric field characteristic flag $e_{flag}$ and temperature characteristic flag $t_{flag}$ are both 1. The physical meaning of this design is that, if and only if an insulator not only deviates significantly from the normal point cloud in the overall feature space, but also the electric field deviation degree and temperature anomaly factor pass their respective statistical and physical double tests, it can be fully convinced that it is a zero-value state caused by insulation failure. The electric field distortion provides the electromagnetic evidence of the decrease in insulation resistance, and the temperature anomaly factor provides the thermodynamic evidence of the weakening of dielectric loss. The two corroborate each other and form a complete physical chain.

When the fusion score exceeds the threshold, but only one of the $e_{flag}$ and $t_{flag}$ is equal to 1, it is determined to be suspicious. This kind of insulator has deviated significantly from the normal distribution in overall statistics, but the anomaly is mainly contributed by a single feature: if only the electric field is abnormal and the temperature is normal, it may be the electric field distortion caused by local pollution, the shielding ring effect, or measurement disturbance; if only the temperature is abnormal and the electric field is normal, it may be that the sheet is on the windward side, which causes local cooling to accelerate, or there is early deterioration but the electric field distortion is not sufficiently evident. Due to the lack of cross-corroboration of the two characteristics, this type of situation should not be directly diagnosed as zero-value, and should be marked as suspicious and a manual review is recommended.

**Figure 10:**
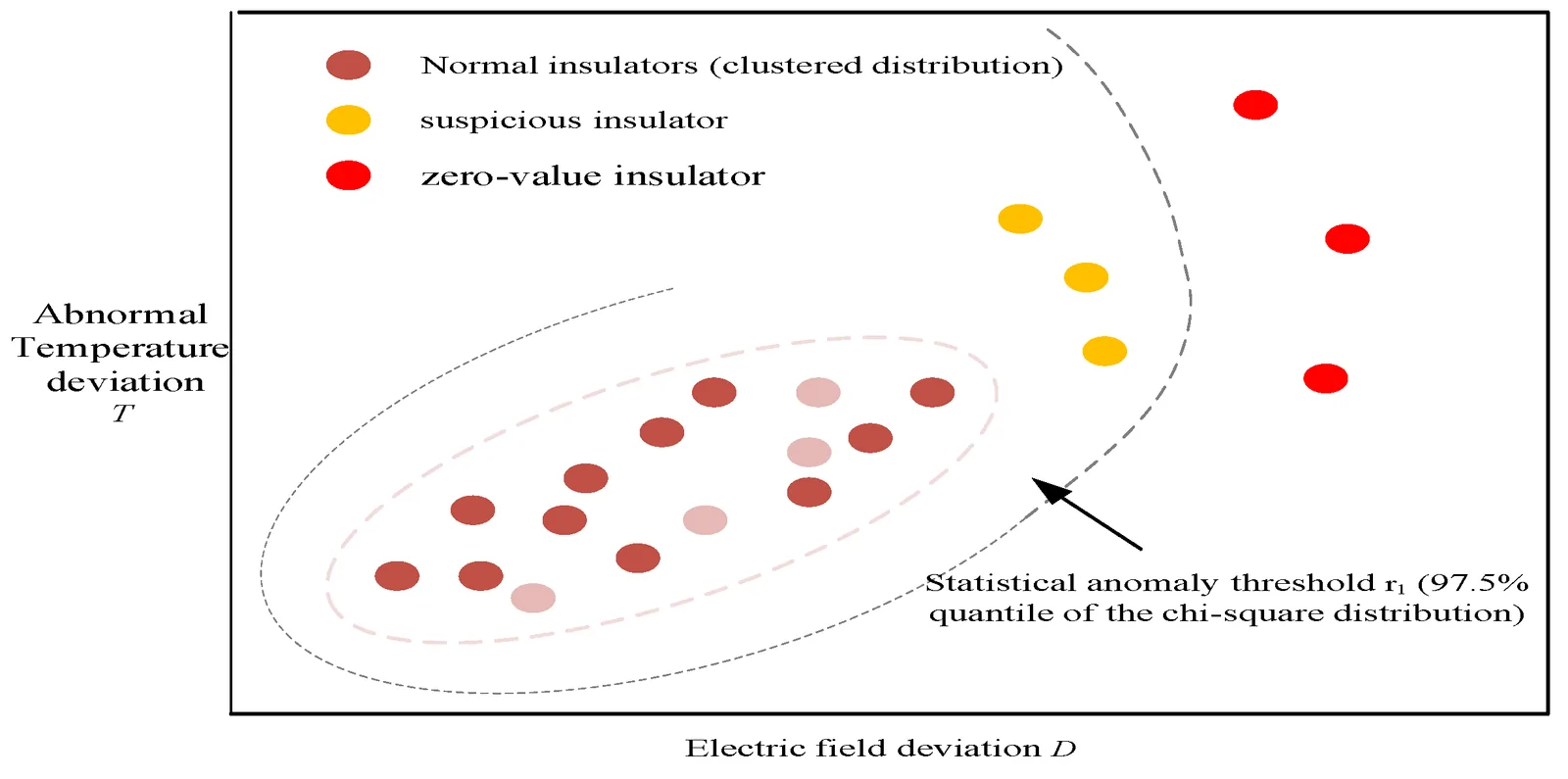
Schematic diagram of the feature space and decision criteria.

If any of the above conditions are not satisfied, it is determined that it is normal. It is particularly noteworthy that, when the statistical threshold is exceeded and both are 0—that is, the Mahalanobis distance shows an anomaly but both eigenvalues are themselves within the physically safe range—this statistical significance stems from a subtle change in the correlation pattern between features or covariance shrinkage caused by a normal subset being too pure, and does not constitute a physical anomaly, so it is still classified as normal. As a specific result, as shown in Figure 10, the setting of this rule effectively suppresses the aforementioned false alarm problem where the “excessive purity of a normal subset causes a small fluctuation to be amplified”.

The grading logic of the above three-level criteria meets the actual needs of transmission inspection: zero-value judgment is used to directly lock insulators to be replaced with high confidence, suspicious judgment provides early warning and a manual intervention buffer for possible abnormalities but insufficient evidence, and normal judgment ensures that most normal insulators are not wrongly disturbed.

## 5. Experimental Validation and Analysis

### 5.1. Experimental Ideas

A sample set of insulator string tests covering multiple working conditions was constructed. The sample includes two structures: 7-piece string and 12-piece string, covering the normal string and the typical positions where the zero value is located at the high-voltage terminal, the middle portion, and the ground terminal, with a total of 129 strings and 1138 insulators. Each insulator is marked with the actual state (normal or zero value) as the criterion for checking the accuracy of the algorithm.

According to the COMSOL electric field simulation method and YOLOv8 infrared temperature extraction method, the composite electric field intensity and surface temperature of each insulator were obtained, and the ambient temperature was recorded at the same time. The normal electric field trend was determined by RANSAC adaptive quadratic fitting method, the electric field deviation degree and temperature anomaly factor were calculated, and the fusion score was obtained by the fusion model. Finally, the state of each insulator was judged by the three-level zero detection criterion.

In order to fully verify the effectiveness of the electric field-temperature fusion zero detection method based on adaptive trend fitting proposed in this paper, several sets of comparative experiments are set up. They are the traditional infrared temperature difference threshold method, single electric field trend method, and SVM three-classification method. The above comparison methods were all evaluated on the same test set as the method in this paper. The parameters of the method in this paper remain unchanged. None of the comparison methods additionally used training data. By comparing the precision rate (Precision), missed detection rate (FNR), false detection rate (FPR), and suspicious rate (SR), the advantages of the proposed method in discrimination accuracy, anti-jamming ability, and engineering prudence are verified.

### 5.2. Experimental Results and Analysis

#### 5.2.1. Evaluation Indicators

In this paper, zero-value insulator detection is set as a three-category problem of normal, suspicious, and zero value; however, ‘suspicious’ is not a positive conclusion, but an early warning signal prompting manual review. If ‘suspicious’ is directly included in the error statistics, two essentially different situations of confusion judgment error and cautious doubt cannot truly reflect the discrimination ability of the algorithm.

Therefore, the following evaluation system is adopted in this paper: the ability of the algorithm to discriminate between normal and zero insulators is evaluated by the definite judgment accuracy rate Precision, missed detection rate FNR, and false detection rate FPR, and the decision prudence of the algorithm is evaluated by suspicious rate SR. Each indicator is defined as follows:

(17) $$\mathrm{Precision}=\frac{TP+TN}{N_{total\text{\_}labeled}}\times 100\%$$

(18) $$\mathrm{FNR}=\frac{FN}{TP+FN+N_{suspicious\text{\_}zero}}\times 100\%$$

(19) $$\mathrm{FPR}=\frac{FP}{FP+TN+N_{suspicious\text{\_}normal}}\times 100\%$$

(20) $$\mathrm{SR}=\frac{N_{suspicious}}{N_{total\text{\_}labeled}}\times 100\%$$

where TP means true positive, that is, the number of tablets that are actually zero and are clearly judged to be zero; TN means true negative, which is the number of tablets that are actually normal and judged to be normal; FP stands for false positive, that is, the number of pieces that are actually normal but misjudged as zero; FN means false negative, that is, the number of pieces that are actually zero but missed as normal; $N_{suspicious\text{\_}zero}$ denotes the number of zero-value units labeled as suspicious; $N_{suspicious\text{\_}normal}$ denotes the number of normal units labeled as suspicious; $N_{suspicious}$ denotes the total number of suspicious units; and $N_{total\text{\_}labeled}$ denotes the total number of insulator units.

#### 5.2.2. Experimental Results

The overall test results using 129 strings of samples are shown in Table 1.

The experimental results show that the accuracy rate reaches 93.8%, which indicates that the proposed method is significantly superior to the other four comparison methods. The infrared temperature difference method is greatly affected by ambient temperature, sunshine, and heat conduction. When the middle part of the string is zero, the missed detection rate is as high as 20.0%, and there are many false detections. Although the single electric field method is sensitive to the zero value of the high voltage terminal and the ground terminal, the deviation degree is not prominent enough when the middle zero value is found, and 15.0% of the detection is missed. The SVM method introduces the suspicious mechanism, but the suspicious rate is as high as 8.3%, including multiple normal insulators marked as suspicious, which increases the unnecessary review workload; at the same time, its definite judgment accuracy is lower than that of this method. This directly verifies the effectiveness of the multiplicative fusion criterion of the “electric field as the main and temperature as the auxiliary”. The method reaches the zero threshold only when two features are significant at the same time, thus effectively suppressing false alarms caused by a single-source anomaly. The missed detection rate of 0.0% indicates that all zero-value insulators have not been missed as normal, of which 120 insulators are directly detected, and 7 insulators are classified as suspicious because of an obvious electric field distortion but the temperature rise does not reach the set threshold, which indicates that manual review is required. In fact, the full coverage of zero-value positions is still achieved. The false detection rate of 0.0% further confirms the reliability of the adaptive trend fitting method in eliminating the zero-value slice interference and establishing the normal electric field benchmark accurately.

The suspicious rate is only 6.2%, and all concentrated on actual zero-value insulators. These seven zero-value insulators are all located in the middle of the insulator string, where the normal electric field value itself is low, and the electric field distortion amplitude caused by a zero value is smaller than that at both ends. At the same time, the heat conduction effect of adjacent normal insulators makes their temperature rise not significantly lower than 2 °C. When facing these samples with insufficient characteristics, the algorithm does not forcibly give a clear judgment of the zero value or normal value, but marks them as suspicious, which reflects the prudence of the three-level criterion design. In the actual inspection, the suspicious mark can prompt the operation and maintenance personnel to conduct a key review of the film, and exchange a very small additional workload for effective control of the risk of a missed inspection.

Based on the above results, the electric field-temperature fusion zero detection method based on the adaptive trend fitting proposed in this paper can accurately identify zero-value insulators on the test samples of 129 strings and 1138 insulators, and all indices reach the expected design goals. At the same time, the effectiveness and robustness of the proposed method under different insulator string structures and zero-value positions are verified.

## 6. Conclusions

In this paper, a complementary zero detection method based on the fusion characteristics of the infrared image and electric field intensity is proposed to solve the engineering problem where a single method easily produces misjudgment and the automation level is insufficient in the zero-value insulator detection of transmission lines. This method takes electric field distortion as the main strategy and temperature anomaly as the supplementary strategy, realizes the automatic extraction of insulator infrared temperature through the YOLOv8 deep learning model, establishes a three-dimensional electric field model of the insulator string by a COMSOL finite element simulation, and designs an adaptive trend fitting and multiplicative fusion algorithm to construct a multi-dimensional criterion matrix. The main conclusions are as follows:

1. A method for constructing the electric field deviation feature based on consensus-based adaptive quadratic fitting is proposed. According to the U-shaped physical law of the electric field distribution of insulator strings, the method introduces the consensus principle of RANSAC and combines it with physical constraints of the normal electric field distribution. Considering the limited number of insulator units in a single string, candidate normal subsets are directly enumerated and evaluated instead of being generated by random sampling. The optimal normal subset is determined according to the inlier consistency and fitting deviation, and the electric field trend line is subsequently fitted to quantify the electric field deviation degree of each insulator. The constructed electric field deviation degree is dimensionless and does not rely on a preset reference database, which effectively overcomes the threshold calibration difficulty caused by differences in electric field amplitude under different voltage levels, insulator models, and detection distances, and provides electric field characteristics with clear physical meaning and strong universality for subsequent fusion criteria.
2. An electric field-temperature fusion diagnosis model based on a robust Mahalanobis distance and a three-level classification criterion are constructed. The electric field deviation and temperature anomaly factors are formed into two-dimensional eigenvectors, and the robust mean and covariance matrix of normal samples are estimated by the minimum covariance determinant algorithm, and the Mahalanobis distance is calculated and normalized into the fusion score. This fusion method adaptively adjusts the weights of each feature direction according to the covariance structure of normal point set, without artificially setting the fusion coefficient, and naturally realizes the effective integration of two kinds of heterogeneous information. On this basis, the characteristic anomaly flag based on median absolute dispersion is introduced, which, together with the statistical significance test, constitutes a double verification mechanism, which effectively avoids the false positive problem caused by the excessive narrowing of the normal subset. The three-level criterion (normal, suspicious, and zero value) not only guarantees the zero-value detection rate, but also introduces a prudent suspicious warning level, which takes into account both the detection sensitivity and decision reliability.
3. Experiments verify the effectiveness and robustness of the method. On 129 strings and 1138 insulator test samples covering 7 strings and 12 strings with zero values at different typical positions, the method achieved the performance of a clear judgment accuracy of 93.8%, missed detection rate of 0.0%, false detection rate of 0.0%, and suspicious rate of only 6.2%. All the insulators that are clearly judged as zero by the algorithm are not misjudged, and all the zero-value insulators are not misjudged as normal. The seven zero-value slices, which are classified as suspicious because of their insufficient characteristics, are all located in the middle of the string with a small electric field distortion amplitude, which meets the physical expectation and reflects the rationality and prudence of the criterion design—the effective control of the risk of missed detection is exchanged for a very small additional review workload.

In summary, the method in this paper organically integrates COMSOL electric field simulation with YOLO infrared temperature extraction, and realizes the collaborative utilization and cross-validation of two kinds of physical features through a robust statistical framework. The results support the feasibility of the proposed decision procedure under the simulation settings and image conditions evaluated here. Its transfer to operating lines with different geometries or environmental boundary conditions requires further validation using measured electric-field data. The follow-up work can be carried out in the following directions: (1) establish a multi-condition electric-thermal benchmark database covering complex environmental conditions such as pollution and high humidity to further enhance the scenario adaptability of the algorithm; and (2) explore the feasibility of replacing simulation data with measured electric field data, reduce the reliance of the method on accurate geometric modeling and material parameters, and expand its application potential in online monitoring and edge computing scenarios.

Future work: In future work, instance segmentation models, such as YOLOv8-seg, will be further investigated to obtain more accurate pixel-level masks of individual insulator units. Compared with the ROI-based processing strategy adopted in this study, pixel-level instance segmentation can more precisely separate the insulator from the surrounding background and is expected to further reduce the interference caused by the sky, vegetation, towers, and conductors, particularly under complex backgrounds or large inclination angles of insulator strings. This may further improve the accuracy and robustness of infrared temperature feature extraction. And it should be noted that actual field measurements may be affected by environmental and operating factors, including the temperature, humidity, contamination, rainfall, wind, and surrounding electromagnetic conditions. The present simulation focuses on establishing the reference electric-field distribution under normal atmospheric conditions and does not attempt to cover all such disturbances. An experimental detection device for electric-field and infrared measurements of insulator strings is currently under development. In subsequent work, measurements obtained using this device will be combined with actual environmental parameters to systematically evaluate the influence of complex operating conditions on the electric-field distribution and diagnostic features. On this basis, environmental compensation or correction strategies will be investigated to further improve the applicability and robustness of the proposed method in practical field environments.

## Figures and Tables

**Table 1:** Experimental Results.

Methods	Total Number of Pieces	Number of Zero-Valued Slices	Correct Determination of Zero Value	Number of Missed Detections	Number of False Detections	Suspicious Rate SR	Clear Judgment Accuracy Precision	Missed Detection Rate FNR	False Detection Rate FPR
Infrared temperature difference threshold method	1138	127	16	25	13	9.9%	89.1%	20%	1%
Single electric field trend method	1138	127	17	19	6	9.6%	89.4%	15%	0.5%
SVM three-classification method	1138	127	114	13	13	8.3%	91.7%	10%	1%
The method of this paper	1138	127	120	0	0	6.2%	93.8%	0%	0%

## Data Availability

The original contributions presented in this study are included in the article. Further inquiries can be directed to the corresponding author.
